# Evaluation of tracer labelled methionine load test in vitamin B-12 deficient adolescent women

**DOI:** 10.1371/journal.pone.0196970

**Published:** 2018-05-24

**Authors:** Dattatray S. Bhat, Lourdes L. Gruca, Carole D. Bennett, Prachi Katre, Anura V. Kurpad, Chittaranjan S. Yajnik, Satish C. Kalhan

**Affiliations:** 1 Kamalnayan Bajaj Diabetology Research Centre, King Edward Memorial Hospital Research Centre, Pune, India; 2 Department of Molecular Medicine, Cleveland Clinic Lerner College of Medicine of Case Western Reserve University, Cleveland, OH, United States of America; 3 Department of Pathobiology, Lerner Research Institute, Cleveland Clinic Foundation, Cleveland, OH, United States of America; 4 St. John’s Research Institute, Bangalore, India; University of Illinois, UNITED STATES

## Abstract

**Background:**

Methionine loading test (MLT) has been used primarily to identify defects in transsulfuration of homocysteine in cystathionine beta synthase deficiency. It may not be as useful to evaluate remethylation pathway, in vitamin B-12 and folate deficiencies.

**Objective:**

We used tracer isotope labelled MLT to interrogate transsulfuration and remethylation independently in vitamin **B-12** deficiency.

**Design:**

We studied vitamin B-12 deficient women with a tracer labelled MLT before and eleven months after treatment with vitamin B-12. The fractional contribution of [^13^C]homocysteine to breath CO_2_ was used as a measure of transsulfuration, and difference in the intracellular enrichment of [^13^C]methionine and that of [C^2^H_3_]methionine as a measure of remethylation of homocysteine. Combined pre- and post-treatment results were analyzed to investigate the association between plasma vitamin B-12 concentrations and measures of homocysteine metabolism.

**Results:**

The subjects were 17 years old, with a BMI of 19.4 kg/m^2^. Treatment with vitamin B-12, 2µg/day increased plasma B-12 from 93 (78.7, 106.2) [median (25^th^, 75^th^ centiles)] to 161.5 (125.5, 226.2) pmol/L; 44% were below <150pmol/L after treatment. Fasting homocysteine concentration was significantly lower and that of cysteine higher in subjects with B-12 levels >150pmol/L. The tracer estimated transsulfuration of homocysteine was lower and remethylation higher with B-12 levels >150pmol/L when compared with those <150pmol/L.

**Conclusions:**

The tracer labelled MLT in combination with fasting parameters is a robust way to estimate parameters of methionine metabolism and can be used in the field where prime-constant rate infusion studies cannot be done efficiently.

## Introduction

Methionine loading test was originally introduced to identify subjects with defects in transsulfuration due to congenital cystathionine beta synthase defects or due to pyridoxine phosphate deficiency. Since then it has been used to identify hyperhomocysteinemia in a number of clinical disorders [1] and in micronutrient deficiency states [2]. It has been suggested that the concentrations of total homocysteine (tHcy) in the plasma during fasting are very sensitive to the abnormality of the remethylation (Rm) of homocysteine while the post methionine load increase in plasma total homocysteine are not as sensitive a measure of the remethylation [3–6]. We postulated that a tracer labelled methionine load, by measuring the remethylation (Rm) and transsulfuration (Ts) directly and independently, will be a robust and informative test for the interrogation of the methionine metabolism in B-12 deficient subjects and other clinical states.

Vitamin B-12 deficiency is common in Indians and is attributed to low intake of foods of animal origin (vegetarian diet) [7–9]. Vitamin B-12 is a cofactor for methionine synthase, which catalyzes the methylation of homocysteine to form methionine. A decrease in the activity of methionine synthase results in lower rates of methylation of homocysteine and high concentration of total homocysteine in the plasma [10]. In an earlier study, we examined the perturbations in markers of one carbon metabolism in multi-nutrient insufficient Indian women and nutritionally sufficient American women [11]. Plasma concentrations of homocysteine were markedly higher in the Indian women, and this was attributable to lower concentrations of vitamin B-12, folate and vitamin B-6 and possibly lower protein intake, even though the plasma concentrations of folate and vitamin B-6 were ‘within the acceptable range’. Oral methionine load showed a greater increase in homocysteine concentration in the Indian subjects compared with the Americans which could be the result of the combined effect of decrease in transsulfuration (primarily) and of remethylation of homocysteine. However, the respective contribution of remethylation and transsulfuration to the observed hyperhomocysteinemia could not be ascertained.

In the present study, we have evaluated the tracer labelled methionine load in adolescent girls who were vitamin B-12 deficient and were being treated with vitamin B-12, to examine the relationship between plasma concentrations of vitamin B-12 and tracer determined measures of methionine metabolism. The data show a strong relationship between plasma vitamin B-12 concentrations and estimates of remethylation and transsulfuration of homocysteine.

## Subjects and methods

The present study was not a clinical trial of B-12 supplement. Study subjects were those excluded from a clinical trial (Pune Rural Intervention in Young Adolescents trial) of vitamin B-12 supplement, for ethical reasons (plasma vitamin B-12 levels <100 pmol/L, -2SD for the population) [12]. Thirty-nine girls agreed to take part in the study. None of these girls were unwell. After the first (visit 1) methionine load procedure, they were prescribed either vitamin B-12 (2 µg/day) alone (n = 19) or vitamin B-12 plus micronutrients (UNIMAPP formula without folic acid) and 20 g milk powder (5 g protein/day) (n = 20). The composition of UNIMAPP formula is listed in S1 Table. The rationale for the oral B-12 with or without supplement was based upon the need to provide care similar to the Pune Rural Intervention in Young Adolescents trial. The oral dose of 2 microgram per day was based upon our pilot study demonstrating adequate response on circulating B-12 levels and plasma homocysteine concentration, in a public health setting [7]. All participants received iron (100 mg elemental iron) and folic acid (500 µg) once a week as per Government of India guidelines [13]. All participants were counseled regarding the need for regular consumption of the supplements, however, adherence was not monitored. The study protocol was reviewed and approved by the ethics committee of the KEM Hospital Research Centre, Pune. After fully explaining the procedure, written informed consent was obtained from one of the parents accompanying the child and each participant gave their assent.

The methionine load test (MLT) was performed before (visit 1) and after eleven months (visit 2) of supplementation. The participants reported to the Diabetes Unit, KEM Hospital the previous evening, and ate dinner in the hospital canteen between 8:00–10:00 PM. Height and weight were recorded by trained staff. In the morning, an indwelling cannula was placed in an ante-cubital vein and kept patent using a “heparin saline lock”. After obtaining a basal (after an overnight fast for 10 hours) blood sample, they received l-methionine 50 mg/kg body weight in orange juice with a low methionine breakfast (estimated methionine content ~58 mg). Breakfast was eaten in less than 20 minutes. Tracer labelled methionine was administered to a subset of nineteen girls (nine taking B-12 alone and ten B-12 plus other supplements). [1-^13^C]Methionine, 99% ^13^C, and [C^2^H_3_]methionine 99% ^2^H, each 2 mg per kg body weight, along with unlabeled l-methionine were administered. Tracer labelled methionine were purchased from Cambridge Isotope Laboratories, Inc., Andover, MA, 01810 USA. Blood samples were obtained for measurement of one carbon related biochemicals and tracer enrichment of plasma methionine and homocysteine at 3 and 5 hours. Breath samples for ^13^C enrichment in expired CO_2_ were collected into vacutainers (Becton Dickenson, NJ, USA) at the same time, and stored at room temperature. Blood samples were centrifuged in the cold and the separated plasma and serum were stored at -80°C for later analysis.

## Laboratory analysis

Hematological parameters were measured on a Beckman Coulter analyzer (Ac·T diff Analyzer, Miami, Florida, USA). Creatinine was measured on an automated biochemistry analyzer (Hitachi 902, Hitachi Corporation, Japan) using a standard kit. Transthyretin was measured using human prealbumin ELISA kit (GenWay Biotech, Inc., San Diego, CA 92121). Inter and intra batch CV for repeated measurements in our laboratory was <8%. Plasma cobalamin (vitamin B-12) was measured by microbiological assay using a colistinsulphate-resistant strain of *L*. *leichmanii* [14]. Plasma folate was measured by microbiological assay using a chloramphenicol-resistant strain of *L*. *casei* [15]. The coefficients of variation for repeated measurements in our laboratory for vitamin B-12 and folate measurements in the plasma were <8%. Vitamin B-6 (Pyridoxal-5-phosphate and Pyridoxal) was measured using commercially available HPLC kit (RECIPE GmbH, Germany) using post column derivatization and fluorescence detector on an automated PerkinElmer 200 series, HPLC instrument (PerkinElmer, 710 Bridgeport Avenue, Shelton, CT 06484 4794, USA). Vitamin B-2 was measured in the whole blood using commercially available HPLC kit (RECIPE GmbH, Germany). The coefficients of variation for repeated measurements in our laboratory for vitamin B-6 and vitamin B-2 measurement were <5%. The concentration of amino acids in plasma was measured by HPLC using an OPA derivative and a fluorescence detector as described [16]. Total homocysteine, cysteine and glutathione concentration in the plasma were measured by reducing the oxidized thiols with sodium borohydride followed by conjugation of the thiols with monobromobimane as described [17]. ^13^C and ^2^H enrichment of methionine and homocysteine in the plasma was measured using GC-mass spectrometry as described previously [18]. ^13^C enrichment of expired CO_2_ was measured at the St. John’s Research Institute, Bangalore, India, using an isotope ratio mass spectrometry instrument (Europa Scientific, Crewe, UK).

## Statistical methods

We checked for the normality of the data and performed descriptive statistics. Data are presented as mean and standard deviation for normally distributed data and as median and 25^th^ and 75^th^ percentiles for skewed data (vitamin B-12). Transthyretin: normal >17.0 mg/dl, mild malnutrition <17.0 and > 10.0 mg/dl and severe malnutrition <10 mg/dl [19]. The incremental (above basal) area under the curve (basal, 3 hour and 5 hour) was computed using trapezoidal rule. Differences between groups were tested by Student’s t-test (paired or unpaired as appropriate), or chi square test or Fishers exact test. Correlation and regression analysis were used to test the associations and size of the effect. Statistical analyses were performed using SPSS 16 (SPSS Inc. Chicago US).

## Results

Of 39 adolescent girls included in this study, 5 did not come for the second visit (3 became pregnant and 2 refused). These five girls were comparable with the rest of the study group with respect to basal plasma vitamin B-12 concentration (median 93.4 vs 93.0 pmol/L). There were no significant differences in the response to supplementation in those who received B-12 alone and those who also received MMN and milk powder (S2 Table). Therefore, we have combined the data for analysis.

Demographic and other characteristics are summarized in Table 1. All subjects were post-pubertal and did not have any health problems. On average these girls were short and thin, 18 (46%) had BMI<18.5 kg/m^2^ (WHO definition for undernourished), and 16 (44%) were anemic (hemoglobin concentration <11.0 g/dL). The concentrations of albumin and creatinine in the plasma were in the normal range. Plasma concentrations of transthyretin (9.8 ± 2.5 mg/dL) were suggestive of moderate protein malnutrition [19]. At follow-up (visit 2) a significant increase in height, weight, and blood hemoglobin concentration was observed. Sixteen (47%) girls continued to have low BMI, only 3 (9%) remained anemic, and there was a reduction in the number with macrocytosis. There was no significant change in the plasma concentration of albumin, total protein and transthyretin.

**Table 1:** Demographic and biochemical characteristics of subjects who completed two visits (n = 34).

	Visit 1 (n = 34)	Visit 2 (n = 34)	P
Age (years)	16.9 ± 0.3	17.9 ± 0.4	0.001
Height (cm)	156.2 ± 5.1	156.4 ± 5.2	0.001
Weight (kg)	47.3 ± 7.8	47.5 ± 6.7	0.019
BMI (kg/m^2^)	19.4 ± 3.1	19.4 ± 2.8	0.108
<18.5—kg/m^2^	18 (46%)	16 (47%)	0.759**
Haemoglobin (g/dL)	11.1 ± 1.0	11.8 ± 0.9	0.001
<11- g/dL	16 (43.6%)	3 (9%)	0.001*
Mean corpuscular volume (fL)	93.6 ± 10.8	86.8 ± 8.0	0.001
Macrocytosis (>100 fL)	13 (30.0%)	3 (8.8%)	0.017*
Microcytosis (<80 fL)	4 (10.3%)	4 (11.8%)	0.645**
Mean corpuscular haemoglobin (pg)	31.7 ± 4.2	30.4 ± 3.3	0.001
Red cell distribution width (%)	15.2 ± 1.7	13.8 ± 1.4	0.001
Creatinine (mg/dL)	0.6 ± 0.1	0.5 ± 0.1	0.001
Albumin (g/dL)	4.2 ± 0.1	4.2 ± 0.1	0.759
Total protein (g/dL)	7.1 ± 0.5	7.1 ± 0.5	0.522
Transthyretin (mg/dL)	9.8 ± 2.5	10.1 ± 2.2	0.727
Vitamin B-12 (pmol/L)	92.0 (78.0, 106.0)	161.5 (125.5, 226.2)	0.001
Vitamin B-12 <150 (pmol/L)	31 (91%)	15 (44%)	0.001**
Folate (nmol/L)	22.9± 15.2	21.4 ± 10.5	0.822
Vitamin B-6 (µg/L)	15.3 ± 13.6	13.5 ± 4.3	0.904
Pyridoxal phosphate (µg/L)	2.1 ± 1.3	1.9± 0.5	0.691
Vitamin B-2 (µg/L)	196.8 ± 22.8	227.6± 53.1	0.005
Homocysteine (µmol/L)	45.1 ± 21.4	19.3 ± 15.8	0.001
Homocysteine > 15 (µmol/L)	34 (100%)	15 (44%)	0.001**
Cysteine (µmol/L)	190.4 ± 35.1	220.3 ± 22.4	0.494
Glutathione (µmol/L)	4.1 ± 1.2	6.1 ±2.4	0.001

Values are mean ± SD, median (25^th^ -75^th^ percentile) or n (%).

P: calculated for 34 subjects who were evaluated at visit 1 and visit 2 by either Students paired t-test,

*Fisher’s exact test or

** chi-square test.

The concentrations of vitamins, homocysteine, cysteine and glutathione in the plasma after an overnight fast are shown in Table 1. Vitamin B-12 concentrations were low prior to supplementation (visit 1) and increased significantly after supplementation (visit 2), though it remained less than 150 pmol/L (deficient) in 15 (44%). The plasma concentrations of folate and vitamin B-6 were within normal range and did not change following supplementation. There was a significant increase in the blood concentration of vitamin B-2.

The concentration of total homocysteine in plasma was high before supplementation and decreased significantly following supplementation. However, 15 (44%) participants continued to have a high concentration of homocysteine after supplementation (>15μmol/L).

The plasma concentration of glutathione increased significantly following B-12 supplementation (Table 1).

### Plasma B-12 and parameters of one carbon metabolism

The present study was not a prospective clinical trial, rather a care protocol where we studied vitamin B-12 deficient subjects, before and after a course of nutrient supplementation to correct vitamin B-12 deficiency. Forty-four percent women continued to have low vitamin B-12 status (plasma vitamin **B-12** <150 pmol/L) probably related to poor adherence. Thus, in order to investigate the relation between plasma B-12 concentration and the parameters of one carbon metabolism, especially the tracer isotopic measurement we pooled pre- and post- supplementation data. We compared the various parameters, in instances where plasma vitamin B-12 concentrations were below 150 pmol/L with those above. These data are presented in Table 2. As shown, the higher vitamin B-12 status was not associated with significant change in concentration of folate, B-6 or B-2. Thus the observed differences could be attributed primarily to the B-12 status. Higher plasma B-12 levels were associated with significantly higher concentration of plasma cysteine, and significantly lower concentration of total homocysteine (Table 2). In addition, there were differences in plasma amino acid concentration: the concentration of serine, histidine, tryptophan, ornithine, citrulline, valine and isoleucine were significantly lower when plasma B-12 levels were >150 pmol/L.

**Table 2:** Plasma concentrations of vitamin B-12, 1-C metabolites and amino acids by vitamin B-12 concentration. Observations were stratified based upon the plasma vitamin B-12 levels more or less than 150 pmol/L (both visit1 and visit2 together).

	B-12 <150 pmol/L (n = 52)	B-12 >150 pmol/L (n = 21)	P
Vitamin B-12 (pmol/L)	96.0 (81.0, 112.5)	218.0 (167.0, 255.0)	0.000
Folate (nmol/L)	17.4 (12.3, 26.2)	21.5 (16.5, 28.8)	0.189
Vitamin B-6 (µmol/L)	13.1 (9.7, 16.3)	14.7 (10.7, 16.2)	0.396
Pyridoxal Phosphate (µmol/L)	1.9 (1.6, 2.1)	2.1 (1.6, 2.4)	0.242
Vitamin B-2 (µmol/L)	202.0 (182.0, 222.5)	199.0 (181.5, 257.2)	0.596
Serine (µmol/L)	173.98 ± 44.09	149.00 ± 39.93	0.03
Histidine (µmol/L)	96.12 ± 15.54	87.48 ± 17.00	0.04
Tryptophan (µmol/L)	40.13 ± 7.82	35.76 ± 5.71	0.02
Ornithine (µmol/L)	66.10 ± 18.29	56.14 ± 16.43	0.03
Citrulline (µmol/L)	34.75 ± 6.31	39.24 ± 6.35	0.01
Valine (µmol/L)	177.10 ± 27.48	160.71 ± 21.01	0.02
Isoleucine (µmol/L)	50.29 ± 7.61	46.52 ± 6.15	0.05
Cysteine (µmol/L)	119.81 ± 34.16	216.64 ± 25.67	0.026
Total homocysteine(µmol/L)	36.60 (24.6, 53.3)	10.70 (9.5, 20.9)	0.000
Glutathione (µmol/L)	4.96 ± 2.01	5.50 ± 2.40	0.377
Incremental AUC of Methionine after Methionine load (µmol/L*300min)	62082.1 ± 14196.2	58075.7 ± 17032.5	0.15
Incremental AUC of homocysteine after Methionine load (µmol/L*300min)	2837.2 (2079.0, 3920.2)	2265.0 (1710.0, 2752.5)	0.021

Values are mean ± SD, or median (25^th^ -75^th^ percentile)

### Response to oral methionine load

Following the oral methionine load, there was an increase in the plasma concentration of methionine which peaked at 3 hour followed by a decrease at 5 hour (S1 Fig). The concentrations of methionine in the plasma at 0 hour, 3 hour and 5 hour and the incremental area under the curve for the plasma methionine response were comparable in the low and high B-12 instances. (Table 2). The concentration of total homocysteine in the plasma significantly increased after the oral methionine load. Total homocysteine concentrations were significantly lower at each time point in instances with plasma B-12 >150 pmol/L (p = <0.001 for all time points) which reflected in the lower incremental area under the curve of plasma homocysteine (p = 0.02). As indicated above, the concentrations of cysteine in the plasma were higher in higher B-12 instances (Table 2). The concentrations of cysteine in the plasma decreased while that of glutathione increased following the methionine load (S1 Fig).

### Responses to tracer labelled methionine

We used [1-^13^C]methionine, and [C^2^H_3_]methionine in order to estimate respectively the changes in transsulfuration and remethylation of homocysteine in relation to B-12 status. The plasma concentration of total homocysteine during fasting was significantly lower in the B-12 sufficient instances (S3 Table).

The responses to oral [1-^13^C]methionine and the contribution of methionine to homocysteine and expired CO_2_ are displayed in Table 3. As shown, the ^13^C enrichment of plasma methionine increased reaching a peak at 3 hour followed by a decrease by 5 hour. The plasma [^13^C]methionine response at 3h and the area under the curve were significantly higher in instances with plasma B-12 >150 pmol/L. The [^13^C] enrichment of homocysteine also was higher reflecting in part the lower homocysteine pool, so that the area under the enrichment curve was higher in these instances. The [^13^C] enrichment of expired CO_2_ is also shown in the table. Plasma B-12 status did not affect the [^13^C] enrichment of CO_2_. The fractional contribution of homocysteine to CO_2_ (^13^CO_2_/^13^Homocysteine ratio) was significantly less in the high B-12 instances, suggesting a lower rate of transsulfuration (Ts) of homocysteine.

**Table 3:** ^13^C enrichment (moles percent excess) of plasma methionine, plasma homocysteine, and expired CO_2_ and calculated fractional contribution of homocysteine to expired CO_2_ (transsulfuration; Ts) following ^13^C labelled methionine load.

	[^13^C]Methionine (%)			[^13^C]Homocysteine (%)			[^13^C]O_2_ (%)			[^13^C]O_2_/[^13^C]Homocysteine (Ts%)		
	3h	5h	AUC	3h	5h	AUC	3h	5h	AUC	3h	5h	AUC
Plasma B-12 (<150 pmol/L) (n = 27)	2.612 ± 0.124	2.209 ± 0.148	524.367 ± 25.3832	1.824 ± 0.239	1.982± 0.161	392.578 ± 43.884	0.0064 ± 0.002	0.0053 ± 0.002	1.2777 ± 0.328	0.355 ± 0.111	0.272 ± 0.088	0.329 ± 0.096
Plasma B-12 (>150 pmol/L) (n = 10)	2.712 ± 0.068	2.253 ± 0.078	542.033 ± 12.502	2.149 ± 0.200	2.153 ± 0.153	451.53 ± 35.543	0.0061 ± 0.001	0.0047 ± 0.002	1.1907 ± 0.242	0.286 ± 0.067	0.219 ± 0.077	0.267 ± 0.066
P	0.005	0.326	0.011	0.001	0.017	0.001	0.60	0.33	0.46	0.049	0.116	0.056

We calculated the intracellular enrichment of the [C^2^H_3_]methionine assuming a dilution of the same magnitude as for [^13^C] tracer as suggested by MacCoss and colleagues [20]. The magnitude of remethylation (Rm) of homocysteine was estimated by the difference in the intracellular enrichment of [^13^C]methionine tracer ([^13^C]homocysteine) and that of [C^2^H_3_]methionine tracer. As shown, the intracellular enrichments of [C^2^H_3_]methionine was significantly higher in the B-12 sufficient instances. In addition, the tracer estimated remethylation of homocysteine ([^13^C]Homocystine -[C^2^H_3_]methionine AUC) was significantly higher in the higher vitamin B-12 instances.

**Fig 1:**
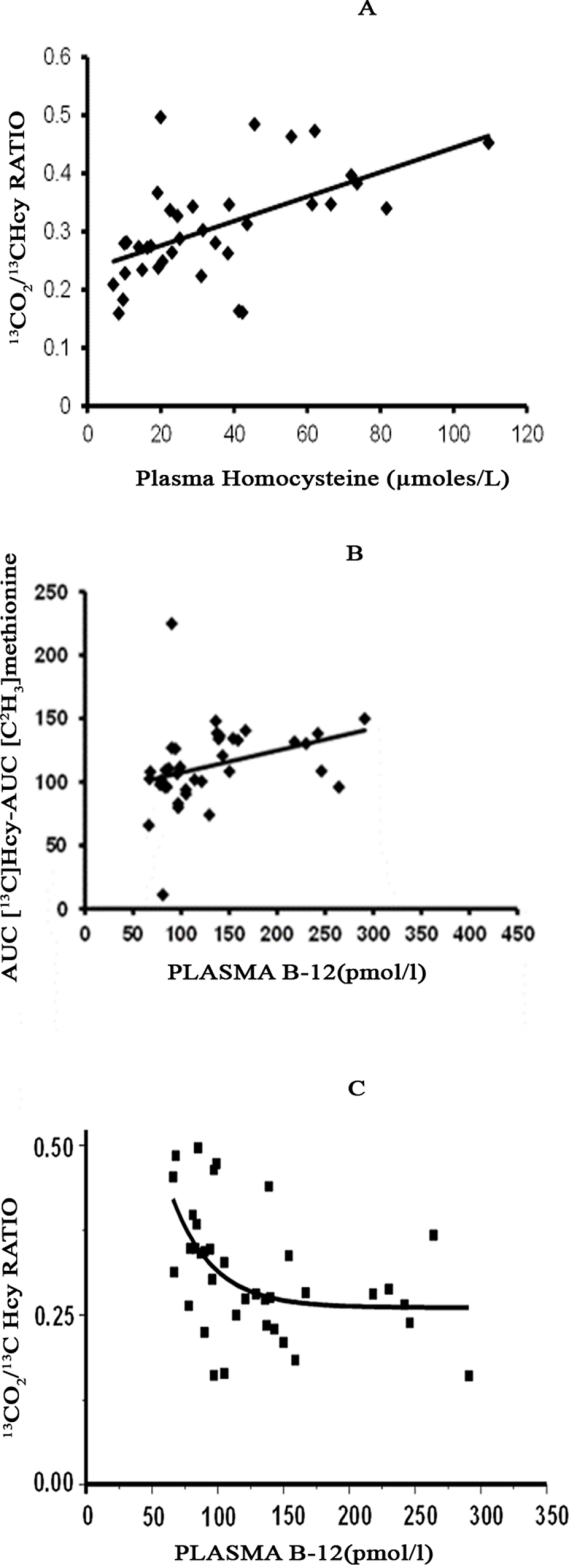
(A) Correlation between basal plasma homocysteine concentration and estimate of transsulfuration (AUC ^13^CO_2_/AUC ^13^CHcy ratio) during methionine load test. Y = 0.002x+0.237, R^2^ = 0.313, P = 0.0003 **(B)** Correlation between basal plasma B-12 levels and estimate of remethylation of homocysteine (AUC [^13^C]Homocysteine–AUC [^2^CH_3_]methionine) during methionine load test. Y = 89.70+0.175*x, P = 0.053. **(C)** Correlation between basal plasma vitamin B-12 concentration and estimates of transsulfuration during methionine load test (y = a-b*c^x: a = 0.261, b = -1.33, c = 0.968, Reduced chi square = 0.007, adjusted R-square = 0.211).

A positive linear correlation was seen between plasma vitamin B-12 concentrations and estimates of remethylation and between plasma homocysteine concentrations and estimates of transsulfuration (Fig 1A and 1B). It is noteworthy that the association between vitamin B-12 and remethylation was direct and linear but did not pass through the 0 coordinate (Fig 1A). In addition, a curvilinear relationship was observed between estimates of transsulfuration and plasma vitamin B-12 concentrations (Fig 1C).

## Discussion

In this study we have evaluated the tracer labelled methionine load to examine the contribution of remethylation and transsulfuration of homocysteine to changes in plasma concentrations of homocysteine and vitamin B-12 in vitamin B-12 deficient adolescent rural Indian girls.

The participants were apparently healthy but short in height, thin and born to undernourished parents, suggestive of chronic ‘life-course’ undernutrition [21]. They were also anemic and had low transthyretin concentrations indicating ongoing micronutrient and protein undernutrition [19]. Dose of vitamin B-12 for supplementation (2 µg/d) was based on recommended dietary allowance (RDA) and on the results of a pilot study [12]. Supplementation improved their vitamin B-12 status as a group, reduced anemia and macrocytosis and plasma total homocysteine concentrations significantly and elevated cysteine and glutathione concentrations. There were no significant effects of supplementation on plasma albumin and transthyretin concentrations.

The present study was not planned as a clinical trial and the intake of supplements by the participants was not supervised or monitored. Vitamin B-12 and micronutrients were given for ethical considerations following exclusion from a clinical trial, because the levels were below < -2SD of the population. Measurements of vitamin B-12 concentrations after supplementation suggest that the adherence to the intake of supplements was variable or the absorption of vitamin B-12 was poor in some. The persistent low transthyretin concentrations suggested no significant change in protein nutrition. The decrease in plasma concentrations of homocysteine during fasting following vitamin B-12 supplementation were anticipated and reflected an improved methionine-homocysteine metabolism. The lower plasma cysteine and glutathione concentrations in the fasting state prior to supplementation suggest a lower transsulfuration, possibly a consequence of lower hepatic S-adenosylmethionine concentrations, an allosteric regulator of transsulfuration. Vitamin B-12 deficiency by decreasing the flux via methionine synthase will decrease remethylation of homocysteine to methionine, would result in lower concentrations of S-adenosylmethionine. These data are in contrast to those seen in response to a methionine load, discussed below, where a higher disposal of pharmacologically induced increase in homocysteine via transsulfuration was observed prior to supplementation. We used tracer labelled methionine to further examine the contribution of remethylation and transsulfuration of homocysteine to the observed responses.

Following methionine load, the plasma concentrations of methionine were not affected by B-12 status suggesting no effect of vitamin B-12 on the absorption of methionine and its disposal from the plasma (S1 Fig). However, the incremental area under the curve (AUC) of plasma homocysteine was significantly decreased in the B-12 sufficient subjects. Since changes in total homocysteine concentration in the plasma are the consequence of its release from the liver, the decrease in its incremental area under the curve would suggest an increase in the intrahepatic metabolism of homocysteine in subjects with B-12 >150 pmol/L, either via remethylation and/or via transsulfuration.

We used [1-^13^C]methionine and the appearance of ^13^C in expired CO_2_ as an index of transsulfuration (2). As anticipated, B-12 status did not have any significant effect upon ^13^C enrichment of methionine in the plasma suggesting no significant change in the metabolism of methionine (Table 3). However, ^13^C enrichment of total homocysteine in the plasma was significantly higher in higher B-12 concentration likely to be the result of decrease in homocysteine pool. The fractional contribution of homocysteine to CO_2_, a measure of transsulfuration was significantly decreased in the B-12 sufficient state. The lower rate of transsulfuration was surprising since change in vitamin B-12 status should not have any direct impact on transsulfuration. We speculate that the observed change is unique to the pharmacological methionine load. Since a fixed amount of methionine load was administered it would result in a corresponding fixed amount of homocysteine being generated. An improved vitamin B-12 status by increasing the remethylation of homocysteine resulted in a decrease in its transsulfuration, underscoring the regulation of methionine cycle at the homocysteine locus [22]. We used the metabolism of [C^2^H_3_]methionine as an index of remethylation of homocysteine [2]. As shown the [C^2^H_3_] enrichment of methionine was significantly more at higher B-12 concentration, in part reflecting the decrease in the homocysteine pool, so that the ratio of enrichments of total homocysteine and methionine was significantly higher in these women. In order to account for the change in the homocysteine pool we calculated the intracellular enrichment of [C^2^H_3_]methionine as suggested by MacCoss and colleagues [20] to estimate the magnitude of remethylation from the difference between intracellular enrichment of [^13^C] and [C^2^H_3_] labelled methionine. As shown in Table 4, the estimate of remethylation of homocysteine was significantly higher when B-12 concentration was >150 pmol/L. These data suggest that following methionine load partitioning of homocysteine towards transsulfuration was decreased as a result of increase in remethylation in subjects with plasma vitamin B-12 greater than 150 pmol/L.

**Table 4:** [C^2^H_3_] enrichment (moles percent excess) of plasma methionine and estimates of remethylation (Rm) of homocysteine folling tracer labelled methionine load in relation to plasma B-12 levels.

	[C^2^H_3_]Methionine (%)			AUC[^13^C]Homocysteine/AUC [^13^C]Methionine	Intracellular [C^2^H_3_]Methionine	AUC[^13^C]Homocysteine-AUC[C^2^H_3_] Methionine (Rm)
	3h	5h	AUC	Ratio	AUC	AUC
Plasma B-12 (<150 pmol/L) (n = 27)	1.937 ± 0.145	1.516 ± 0.323	381.46 ± 37.29	0.748 ± 0.062	285.32 ± 36.93	107.26 ±35.85
Plasma B-12 (>150 pmol/L) (n = 10)	1.971 ± 0.084	1.563 ± 0.079	389.43 ± 16.94	0.839 ± 0.069	326.47 ± 28.33	127.16 ± 16.94
p	0.38	0.49	0.38	0.002	0.002	0.029

There was a significant linear correlation between plasma B-12concentration and the estimates of remethylation (Fig 1). Since vitamin B-12 is a cofactor for methionine synthase, a linear relationship is not surprising. It is significant that the correlation trendline did not cross the ordinate at zero suggesting the contribution of factors other than B-12 to remethylation of homocysteine. We posit that these factors may be the contribution of betaine homocysteine methyl transferase [2] or the impact of low dietary protein intake as suggested by low transthyretin concentrations in our study subjects [19]. Although not measured, dietary betaine intake could be high in the vegetarian populations [23] and could potentially compensate for the impaired remethylation due to poor vitamin B-12 or folate status. Studies of betaine homocysteine methyl transferase (BHMT) deleted mice suggest a significant contribution of betaine to remethylation of homocysteine at least in the rodent [24]. In addition vitamin B-12 folate mediated remethylation of homocysteine did not compensate for the loss of remethylation in the BHMT deleted mice [24, 25]. It may also explain the maintenance of creatinine concentration and the lower incidence of clinical symptoms particularly in the younger vitamin B-12 deficient subjects [26]; however such an inference requires carefully conducted studies in the future. The linear correlation between homocysteine concentration and its fractional contribution to CO_2_ (Ts) is consistent with the known allosteric regulation of Ts (cystathionine beta synthase) by SAM. A relatively high level of SAM following methionine load in low vitamin B-12 state, and therefore low remethylation rate, will result in increased transsulfuration of homocysteine.

In summary, we have evaluated the use of tracer labelled methionine load to examine the partitioning of homocysteine in the transsulfuration and remethylation pathways. We took advantage of an ongoing clinical supplementation of a group of adolescent girls who were diagnosed to have low vitamin B-12 status. Since vitamin B-12 is a cofactor for methionine synthase and thus contributes to remethylation of homocysteine, the clinical study group provided us with a unique opportunity to examine whether the tracer labelled methionine load test can be used to interrogate transsulfuration as well as remethylation of homocysteine. The present data provide important insights into changes in homocysteine metabolism as a result of vitamin B-12 insufficiency. Our data show that the tracer labelled methionine load, in combination with the fasting data, is a robust and useful way to estimate parameters of one carbon metabolism and can be used in the field where prime-constant rate infusion studies cannot be done efficiently. Having validated it in vitamin B-12 deficient subjects, now it can be employed for studies of physiological changes in one carbon metabolism in nutritionally sufficient states such as pregnancy in humans [18] and examine their relationship with fetal growth and the potential epigenetic influences on the health of the offspring.

## Supporting information

S1 Datasheet Data used for the analysis in the manuscript. (XLS)

S1 Fig Concentrations of methionine, homocysteine, cysteine and glutathione in the plasma during methionine load test, pre- (♦) and post- supplementation (□). After an overnight fast, each subject received methionine 50 mg/kg mixed with orange juice. All data are shown in µmol/L and by mean (SD). Incremental area under the curve (AUC) for methionine and homocysteine are shown as bar diagram. Difference between pre- and post- supplementation is shown by * sign; *p<0.05, **p<0.01, ***p<0.001. Difference from basal at 3h and 5h is shown by + sign; +p<0.05, ++p<0.01, +++p<0.001.
(DOCX)

S1 Table Contents of the multinutrient mixture and milk powder supplements. (DOC)

S2 Table Demographic and biochemical characteristics of study subjects in two groups (median and 25th-75th centile). (DOCX)

S3 Table Plasma concentrations of vitamin B-12 and 1-C metabolites by vitamin B-12 concentration in women who underwent tracer labelled methionine load test. Observations were stratified based upon the plasma vitamin B-12 levels more or less than 150 pmol/L (both visit1 and visit2 together).
(DOCX)
