# Supplementary material for: Evaluation of tracer labelled methionine load test in vitamin B-12 deficient adolescent women
Source: PLoS One. 2018 May 24;13(5):e0196970. doi: 10.1371/journal.pone.0196970 (PMC5967743; doi:10.1371/journal.pone.0196970)
Supplement: S1 Fig — After an overnight fast, each subject received methionine 50 mg/kg mixed with orange juice. All data are shown in µmol/L and by mean (SD). Incremental area under the curve (AUC) for methionine and homocysteine are shown as bar diagram. Difference between pre- and post- supplementation is shown by * sign; *p<0.05, **p<0.01, ***p<0.001. Difference from basal at 3h and 5h is shown by + sign; +p<0.05, ++p<0.01, +++p<0.001. (DOCX) [file pone.0196970.s002.docx]

**S1 Fig: Concentrations of methionine, homocysteine, cysteine and glutathione in the plasma during methionine load test, pre- (♦) and post- supplementation (□). After an overnight fast, each subject received methionine 50 mg/kg mixed with orange juice. All data are shown in µmol/L and by mean (SD). Incremental area under the curve (AUC) for methionine and homocysteine are shown as bar diagram. Difference between pre- and post- supplementation is shown by * sign; *p<0.05, **p<0.01, ***p<0.001. Difference from basal at 3h and 5h is shown by + sign; +p<0.05, ++p<0.01, +++p<0.001.**
