# Supplementary material for: Evaluation of tracer labelled methionine load test in vitamin B-12 deficient adolescent women
Source: PLoS One. 2018 May 24;13(5):e0196970. doi: 10.1371/journal.pone.0196970 (PMC5967743; doi:10.1371/journal.pone.0196970)
Supplement: S1 Table — (DOC) [file pone.0196970.s003.doc]

S1 Table. Contents of the multi nutrient mixture and Milk powder supplements.

| Content | Quantity/day |  |  |
| --- | --- | --- | --- |
| **Vitamin B-12 capsule** |  |  |  |
| Vitamin B-12 | 2.0 µg |  |  |
|  | | | |
| **MMN capsule** |  | **Milk powder composition** | **Quantity/day** |
| Vitamin B-12 | 2.0 µg | Dose | 20 gm |
| Vitamin A | 600 µg | Composition |  |
| Vitamin D | 200 IU | Protein | 5.0 gm |
| Vitamin E | 10 mg | Total Carbohydrate | 11.4 gm |
| Vitamin C | 40 mg | Fat | 0.0 gm |
| Vitamin B-1 | 1.5 mg | Energy | 65 Kcal |
| Vitamin B-2 | 1.8 mg |  |  |
| Vitamin B-3 | 20 mg |  |  |
| Vitamin B-6 | 1.0 mg |  |  |
| Zinc | 12 mg |  |  |
| Copper | 2.0 mg |  |  |
| Selenium | 40 µg |  |  |
| Iodine | 150 µg |  |  |
