# Supplementary material for: Evaluation of tracer labelled methionine load test in vitamin B-12 deficient adolescent women
Source: PLoS One. 2018 May 24;13(5):e0196970. doi: 10.1371/journal.pone.0196970 (PMC5967743; doi:10.1371/journal.pone.0196970)
Supplement: S2 Table — (DOCX) [file pone.0196970.s004.docx]

S2 Table. Demographic and biochemical characteristics of study subjects in two supplemental groups (median and 25^th^-75^th^ centile).

|  | B12 group | | MMN group | |  |
| --- | --- | --- | --- | --- | --- |
|  | Pre- Intervention n=19 | Post-intervention n=18 | Pre- Intervention n=20 | Post-intervention n=16 | P |
| Age (y) | 16.9 (16.5, 17.3) |  | 16.9 (16.6, 17.3) |  |  |
| Height (cm) | 155.8 (152.8, 159.1) | 155.9 (153.0, 160.1) | 156.7 (151.4, 161.3) | 156.5 (151.1, 161.7) | NS |
| Weight (kg) | 44.9 (40.8, 49.5) | 45.1 (41.1, 52.8) | 47.6 (42.9, 53.9) | 47.0 (45.0, 53.7) | NS |
| BMI (kg/m^2^) | 18.3 (16.6, 21.1) | 18.1 (16.3, 21.6) | 19.5 (17.8, 21.4) | 19.5 (18.2, 21.5) | NS |
| Haemoglobin (gm/d) | 11.1 (10.5, 12.1) | 11.9 (11.4, 12.3) | 11.3 (10.5, 11.5) | 11.6 (11.1, 12.6) | NS |
| Creatinine (mg/d) | 0.6 (0.5, 0.6) | 0.6 (0.5, 0.7) | 0.6 (0.5, 0.7) | 0.6 (0.5, 0.7) | NS |
| Albumin (gm/d) | 4.2 (4.1, 4.3) | 4.2 (4.1, 4.3) | 4.2 (4.0, 4.4) | 4.3 (4.2, 4.5) | NS |
| Total protein (gm/d) | 7.0 (6.7, 7.4) | 6.9 (6.7, 7.1) | 7.2 (6.6, 7.5) | 7.2 (6.9, 7.4) | NS |
| Transthyritin (mg/d) | 9.5 (7.8, 12.4 | 9.9 (8.1, 11.5) | 9.4 (8.0. 10.8 | 10.4 (8.8, 13.1) | NS |
| Vitamin B-12 (pmol/L) | 89.0 (76.0, 106.0) | 167.0 (125.0, 234.0) | 95.0 (80.2, 105.0) | 154.5 (117.7, 215.2) | NS |
| Folate (nmol/L) | 19.0 (12.2, 32.7) | 18.6 (14.6, 25.3) | 16.4 (13.4, 25.6) | 21.4 (11.7, 28.1) | NS |
| Homocysteine (µmol/L) | 41.5 (26.2, 66.4) | 14.5 (9.9, 26.4 | 41.3 (27.2, 51.4) | 11.6 (9.7, 20.1) | NS |
| Cysteine (µmol/L) | 194.5 (170.0, 218.5) | 227.0 (215.2, 240.0) | 185.0 (159.8, 226.2) | 207.5 (192.7, 235.5) | NS |
| Glutathione (µmol/L) | 3.6 (3.0, 4.7) | 6.4 (5.0, 9.5) | 4.1 (3.8, 5.1) | 4.8 (4.2, 6.9) | NS |

P refers to the significance of difference in the post intervention measurements in the two groups
