# Supplementary material for: Evaluation of tracer labelled methionine load test in vitamin B-12 deficient adolescent women
Source: PLoS One. 2018 May 24;13(5):e0196970. doi: 10.1371/journal.pone.0196970 (PMC5967743; doi:10.1371/journal.pone.0196970)
Supplement: S3 Table — Observations were stratified based upon the plasma vitamin B-12 levels more or less than 150 pmol/L (both visit1 and visit2 together). (DOCX) [file pone.0196970.s005.docx]

S3 Table: Plasma concentrations of vitamin B-12 and 1-C metabolites by vitamin B-12 concentration in women who underwent tracer labelled methionine load test. Observations were stratified based upon the plasma vitamin B-12 levels more or less than 150 pmol/L (both visit1 and visit2 together).

|  | **B-12 <150 pmol/L**  **(n=27)** | **B-12 >150 pmol/L**  **(n=10)** | **P** |
| --- | --- | --- | --- |
| **Vitamin B-12 (pmol/L)** | 96.5 (83.2, 127.0) | 230.0 (163.0, 255.0) | 0.000 |
| **Cysteine (µmol/L)** | 202.1 ± 31.1 | 218.1 ± 27.2 | 0.09 |
| **tHcy (µmol/L)** | 40.3 ± 24.8 | 16.5 ± 6.7 | 0.004 |
| **Incremental AUC of Methionine after Methionine load (µmol/L*300min)** | 69612.9 ±11536.7 | 74040.0 ± 9111.8 | 0.15 |
| **Incremental AUC of tHcy after Methionine load (µmol/L*300min)** | 3140.6 ± 1155.8 | 2930.1 ± 1412.2 | 0.33 |

Values are mean ± SD, or median (25^th^ -75^th^ percentile)
